# Prevalence and outcomes of delirium in community and non-acute care settings in people without dementia: a report from the Canadian Study of Health and Aging

**DOI:** 10.1186/1741-7015-4-15

**Published:** 2006-06-23

**Authors:** Melissa K Andrew, Susan H Freter, Kenneth Rockwood

**Affiliations:** 1 Division of Geriatric Medicine, Dalhousie University, Halifax, NS, Canada

## Abstract

**Background:**

While delirium is common among older adults in acute care hospitals, its prevalence in other settings has been less well studied. We examined delirium prevalence and outcomes in a large cohort of older Canadians living outside of acute care.

**Methods:**

In this secondary analysis of the Canadian Study of Health and Aging, the prevalence of clinically diagnosed delirium was estimated and five-year survival was compared with that of individuals with dementia of graded severity.

**Results:**

Delirium was very uncommon (prevalence <0.5%) and was associated with reduced survival, similar to that of moderate-to-severe dementia.

**Conclusion:**

In this cohort of older Canadians, delirium in non-demented people was associated with very low 5-year survival, at levels comparable with advanced dementia. Although it is common in hospital, delirium is uncommon among older adults in their usual place of residence, suggesting that it is a potent stimulus to seek medical care.

## Background

Delirium is common among older adults in acute care settings (prevalence estimates typically range from 10–60% [1]) and is associated with poor outcomes [2-6]. Few studies, however, have investigated either the prevalence or the outcomes of delirium in non-acute care settings [7-9].

Study of outcomes following delirium diagnosed in non-demented older adults in their usual place of residence outside of acute care is of particular interest. Poor outcomes in such cases would suggest that delirium may be a marker of increased frailty and susceptibility to perturbation of a delicately held balance (even in those with a strong cognitive baseline), rather than a transient and fully reversible condition associated only with acute insults [10,11].

Our aim was twofold: (1) to determine the prevalence of clinically diagnosed delirium in people without dementia who resided in community and long-term care settings, and (2) to compare 5-year outcomes in people with and without delirium.

## Methods

### Study sample

The Canadian Study of Health and Aging (CSHA) is a representative study of dementia and related conditions in older Canadians (age ≥ 65) [12]. The sample was clustered within 5 regions and stratified by age, with over-sampling of those aged 75 and older. Baseline assessments (CSHA-1) on 10,263 individuals were conducted in 1991, of whom 2,914 had a full clinical examination. Examinations were conducted on all 1,255 individuals in long term care facilities, as well as community-dwellers who screened positive for cognitive impairment (defined as a Modified Mini-Mental State Examination score of <78) [13], and a random sample without impaired cognition. As such, the CSHA clinical sample used in this study is enriched for cognitive impairment and frailty [11,12]. Follow-up at 5 (CSHA-2) and 10 (CSHA-3) years included repeat clinical assessments for these 2,914 individuals. Clinical examinations were also undertaken on people with incident cognitive impairment between study waves and on a second random sample without cognitive impairment [12]. Here we include all participants who had a clinical assessment in CSHA-1 and/or CSHA-2 (1,658 community-dwellers and 1,672 residents of long-term care institutions).

### Measures

Delirium was diagnosed using DSM-III-R criteria at a case conference following assessments by a nurse and physician and an independent neuropsychological evaluation. Delirium and dementia diagnoses were mutually exclusive, so no individual with dementia would have been given a final study diagnosis of delirium [12]. In cases of co-existing delirium and dementia (*i.e*. delirium superimposed on dementia), dementia diagnoses took precedence and study participants were classified as having dementia not delirium. Dementia severity was graded using the Global Deterioration Scale (GDS) [12] as mild (GDS = 4) moderate (GDS = 5) or severe (GDS = 6). Functional impairment was necessarily assayed differently in the community and institutional settings, so that urinary continence and the need for assistance with dressing were the only items relevant in both settings that had sufficient variability for analysis.

### Analysis

Characteristics of individuals diagnosed with delirium were compared with those with No Cognitive Impairment (NCI), with those who had other forms of Cognitive Impairment but No Dementia (CIND), and with those with dementia by stage. Fisher's exact test was used to analyze differences in proportions between individuals with and without delirium. Survival following delirium was compared with that of individuals without cognitive impairment and with dementia of graded severity. We graded dementia severity to contextualize better the extent to which the worse outcomes that might be expected with delirium compare with other relevant states. Sampling weights were available to take into account the complex sampling methodology of CSHA-1, and were used in deriving prevalence estimates from CSHA-1. No sampling weights were available for CSHA-2, a survival cohort.

### Ethics

Ethical approval was obtained from the ethics committees of each of the 18 study centres. Written, informed consent for participation in the CSHA was obtained from each participant or proxy respondent. This secondary analysis was approved by the Research Ethics Committee of Capital District Health Authority, Halifax, Canada.

## Results

Delirium (without a history of dementia) was diagnosed in 10 individuals at CSHA-1 and 11 at CSHA-2. Population prevalence at baseline was low (<0.5%). Of these 21 non-demented individuals with delirium at either CSHA-1 or CSHA-2, 12 were community-dwelling and 9 lived in long-term care facilities. Prevalence of delirium in community-dwellers did not differ from that of long-term care facility residents (p = 0.28). Mean age at diagnosis was 86.8 (SD = 6.6) years, and 11/21 (52%) were male.

**Figure 1:**
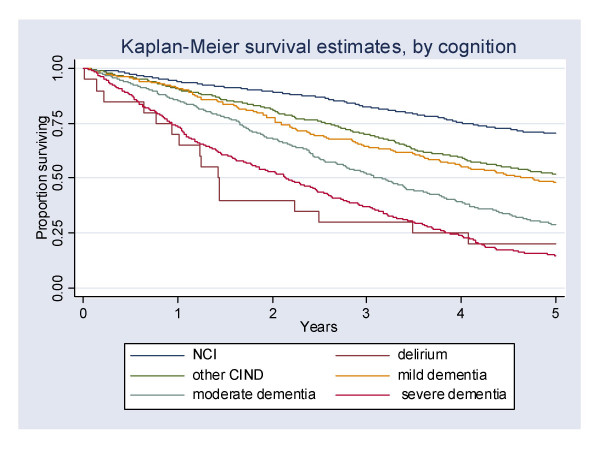
Kaplan-Meier survival curve illustrating five-year survival in delirium and cognitive impairment of graded severity. NCI = No cognitive impairment, CIND = Cognitive impairment, no dementia. Dementia severity was graded using the Global Deterioration Scale (GDS) as mild (GDS = 4) moderate (GDS = 5) or severe (GDS = 6) [12].

Four of the 21 individuals remained alive at five-year follow-up. Five-year survival was lower in those who had been diagnosed with delirium than in the rest of the CSHA cohort (18% vs. 70%, p < 0.001), and there was a trend towards lower survival among individuals with delirium than that seen (53%) in the cohort of individuals who underwent detailed clinical examination (a group that is enriched for medical illness and cognitive impairment owing to the study methodology [11,12] (p = 0.09). Survival is illustrated in Figure 1. Among the 17 individuals with delirium who died, mean survival time was 545 (SD = 410) days following the clinical diagnosis. Of the 4 survivors, 3 lived in long term care facilities at the time of follow-up; 2 of these had been living in the community at baseline and were thus incident institutionalizations.

Individuals with delirium (but no underlying dementia) were older than people with no cognitive impairment, other forms of CIND and all stages of dementia except severe (Table 1). As expected, their Modified Mini-Mental State Examination (3MS) scores were worse than those of people with NCI, but better than those of individuals with moderate and severe dementia. Functional impairment had been present for less time than in moderate and severe dementia. Prevalence of urinary incontinence was high among individuals with delirium (70%, 95% CI: 48–92%) and was higher than that seen in all groups except severe dementia. Survival was worse in delirium (18%; 95% CI:0.7–36%) than in other non-demented conditions and similar to that seen in people with moderate (26%; 95% CI:22–30%) to severe (12%; 95% CI: 9–15%) dementia.

This finding of a survival difference was robust to adjustment for age and sex using a Cox proportional hazards approach. Survival in moderate dementia (HR 0.79, 95% CI: 0.47–1.30, p = 0.35) and severe dementia (HR 1.24, 95% CI: 0.75–2.06, p = 0.40) was similar to that seen in delirium. Mortality in the other states was statistically significantly lower than that seen in delirium: NCI (HR 0.26, 95% CI:0.15–0.43, p < 0.001), other forms of CIND (HR 0.45, 95% CI:0.27–0.74, p = 0.002), and mild dementia (HR 0.49, 95% CI:0.30–0.82, p = 0.007).

## Discussion

We found that delirium was very uncommon (< 0.5%) in this population of older Canadians without dementia living in their usual place of residence. Delirium had a notably poor outcome, with a 5-year survival comparable with advanced dementia.

Our findings must be interpreted with caution. Although the CSHA sample was large, the number of individuals diagnosed with delirium (21) was small, resulting in wide confidence intervals. The small number of cases and resulting wide confidence intervals serve to illustrate an important point, which is that delirium is very uncommon outside acute care settings.

Delirium is often superimposed on dementia. A recent study of community-dwelling older adults in a managed care organization administrative database in the United States found that 13% of older adults with dementia had superimposed delirium [14]. In focusing solely on delirium diagnosed in the absence of underlying dementia, our study probably underestimates the true prevalence of delirium in community-dwelling older adults. This is especially true given that the prevalence of dementia in residents of Long Term Care in this CSHA sample is high (64%) and that only delirium cases occurring among the remaining 36% would have been included in our study. The direction of bias in our study, in which cases of delirium superimposed on dementia were not counted as delirium, probably resulted in a more conservative comparison of outcomes, given that individuals with dementia underlying their delirium might reasonably be expected to have poorer outcomes than individuals who were previously cognitively intact. Including these individuals in the delirium group might therefore have led to a finding of even poorer outcomes in comparisons of delirium with dementia of graded severity.

As in other studies, the diagnosis of both delirium and dementia was clinical, although the use of multiple observers and preliminary and final diagnostic opinions at a case conference are important aids to judgment. In particular, diagnosis of dementia relied on comprehensive clinical and neuropsychological assessments and not on a history of previously diagnosed dementia.

The finding that cognitive function, as measured by the 3MS, was better than that seen in moderate and severe dementia, yet survival was not, provides valuable new information. The prevalence of urinary incontinence among individuals with delirium was higher than that observed in all cognitive subgroups except severe dementia. This may reflect the association of delirium with underlying frailty and medical comorbidity [2]. In contrast, individuals with delirium (but no underlying dementia) had duration of functional (dressing) impairment similar to those with NCI, CIND and mild-moderate dementia, but shorter than those with severe dementia, probably as a consequence of their better pre-morbid cognitive function.

Delirium is known to be common and associated with acute illness in older patients presenting to and admitted to acute care facilities [1-5]. One might expect delirium in older adults outside of acute care to be associated with high mortality in the short term owing to underlying acute illness that is not being treated. While 5-year mortality in the 21 patients identified in this study was high, several individuals survived hundreds of days following their diagnosis, so their 5-year mortality was not driven by deaths immediately following the study assessment. This suggests that while delirium operates as a marker of frailty (and thus increases susceptibility), overall lethality commonly depends also on an accumulated burden of deficits [11].

Few studies have investigated delirium outside of acute care. One study found that 10% of a sample of 199 older adults without dementia aged 85+ who were community-dwelling and free of dementia and delirium at baseline developed an episode of delirium over a 3 year follow-up period, and that these individuals had higher mortality than those who had not developed delirium [8]. Our results are consistent in that we found increased 5-year mortality in those diagnosed with delirium, though we employed a point-prevalence design rather than studying incidence of delirium. The 1981 community-based Eastern Baltimore Mental Health Survey clinically diagnosed delirium in 6 of 810 adults; the prevalence was 1.1% in adults over age 55 and 13.6% in the > = 85 age group [7]. We found a much lower prevalence of delirium (<0.5%) even among subjects aged 85 and over. Caution must be employed in considering this comparison in view of the small numbers of cases in both studies: the 13.6% prevalence was based on a single case of delirium in a sub-sample of 16 individuals aged 85 and over [7]. Although our prevalence estimate is also based on a small number of cases, our denominator is much larger. A Swedish prevalence study involving older adults in various care settings found a high prevalence of delirium: 58% in nursing homes, 35% in old people's homes and 35% in older people living in their own homes with home care services [9]. The prevalence in our study population of adults aged ≥ 65 years was much lower. One possible explanation is that our study was population-based, whereas in the Swedish study the prevalence of dementia was high and all the older adults included were receiving care services and thus represented a population that was more frail (and at higher risk of delirium) than ours. In the CSHA, clinical diagnoses of delirium and dementia were mutually exclusive. Given that cognitive impairment and dementia are established risk factors for delirium [8,15], one would expect lower prevalence of delirium in non-demented older adults.

## Conclusion

Delirium is uncommon among non-demented older adults in their usual place of residence, suggesting that it is a potent stimulus to seek medical care. Even in the absence of dementia, delirium is associated with low 5-year survival, at levels comparable with advanced dementia.

## Competing interests

The author(s) declare that they have no competing interests.

## Authors' contributions

KR is a principal investigator in the CSHA. All three authors participated in the design of the analyses. MKA did the analyses and wrote the first draft of the paper. SHF and KR reviewed the analyses and revised the manuscript. All authors read and approved the final manuscript.

## Pre-publication history

The pre-publication history for this paper can be accessed here:



## Figures and Tables

**Table 1:** Characteristics and survival of individuals diagnosed with delirium compared with no cognitive impairment (NCI), other forms of cognitive impairment, no dementia (CIND) and graded severities of dementia. Dementia severity was graded using the Global Deterioration Scale (GDS) as mild (GDS = 4) moderate (GDS = 5) or severe (GDS = 6) [12].

	**Delirium** **N = 21**	**NCI** **N = 921**	**Other CIND** **N = 851**	**Mild dementia** **N = 253**	**Moderate dementia** **N = 441**	**Severe dementia** **N = 421**
**Age** **(95% CI)**	86.8 (83.8, 89.9)	79.0 (78.5, 79.4)	80.2 (79.8, 80.7)	82.1 (81.2, 82.9)	82.8 (82.1, 83.5)	84.1 (83.4, 84.9)
**% female mean** **(95% CI)**	47.6 (24.3, 70.9)	61.7 (58.5, 64.8)	61.5 (58.2, 64.7)	60.9 (54.8, 66.9)	65.5 (61.1, 70.0)	77.4 (73.4, 81.4)
**Education mean years** **(95% CI)**	9.7 (8.3, 11.2)	9.3 (9.0, 9.5)	8.1 (7.8, 8.4)	8.4 (7.9, 8.9)	8.3 (7.9, 8.7)	8.3 (6.7, 9.8)
**3MS mean** **(95% CI)**	68.1 (58.9, 77.2)	84.7 (84.0, 85.3)	71.9 (70.9, 72.9)	63.4 (61.7, 65.1)	47.3 (45.7, 48.9)	18.3 (16.3, 20.4)
**Months with impaired dressing mean** **(95% CI)**	3.9 (0, 9.6)	1.0 (0.4, 1.6)	3.1 (2.0, 4.3)	5.0 (2.5, 7.5)	10.3 (7.8, 12.8)	25.6 (22.3, 28.8)
**Urinary incontinence %** **(95% CI)**	70.0 (48.0, 92.0)	17.0 (14.5, 19.4)	25.6 (22.7, 28.6)	27.0 (21.5, 32.6)	44.9 (40.2, 49.7)	84.8 (81.1, 88.6)
**5-year survival %** **(95% CI)**	18.2 (0.7, 35.7)	69.6 (66.6, 72.6)	51.4 (48.0, 54.9)	46.2 (39.9, 52.4)	26.0 (21.8, 30.1)	12.2 (9.0, 15.3)
